# Antitumor Triterpenoid Saponin from the Fruits of *Avicennia marina*

**DOI:** 10.1007/s13659-018-0167-9

**Published:** 2018-05-25

**Authors:** Xiong-Wu Yang, Zhi Dai, Bei Wang, Ya-Ping Liu, Xu-Dong Zhao, Xiao-Dong Luo

**Affiliations:** 1 State Key Laboratory of Phytochemistry and Plant Resources in West China, Kunming Institute of Botany, Chinese Academy of Sciences, Kunming, 650201 People’s Republic of China; 2 Key Laboratory of Animal Models and Human Disease Mechanisms, Kunming Institute of Zoology, Chinese Academy of Sciences, Kunming, 650223 People’s Republic of China; 3 University of Chinese Academy of Sciences, Beijing, 100049 People’s Republic of China; 4 Yunnan Key Laboratory of Natural Medicinal Chemistry, Kunming, 650201 People’s Republic of China

**Keywords:** *Avicennia marina*, Triterpenoid saponin, GSCs, DPPH

## Abstract

The fruits of *Avicennia marina* are widely used for both medicine and food in Guangxi of China. As a part of our continuous effort to search for bioactive molecules from the plant, the fruits of *A. marina* were investigated, which has led to one new triterpenoid saponin (**1**) and 29 known compounds been isolated and their structures were established by using spectroscopic methods and comparing with literature data. The new triterpenoid saponin showed cytotoxicity against GSC-3# and GSC-18# with the IC_50_ values were 12.21 and 5.53 μg/mL respectively, and most of the known compounds had significant antioxidant capacity with the IC_50_ values ranging from 0.36 to 13.07 μg/mL.

**Electronic supplementary material:**

The online version of this article (10.1007/s13659-018-0167-9) contains supplementary material, which is available to authorized users.

## Introduction

Functional foods not only provide variously essential nutrition to body, but also be benefit for preventing diseases and maintaining health [1, 2]. These foods especially have multiple bioactivities, such as antioxidant, anti-inflammatory, antibacterial and anticancer activities [3, 4], so people can benefit from daily consumption of them to keep from or relieve the occurrence of chronic age-related diseases or life-style diseases [5, 6]. With the benefits, people have paid more and more attention to these foods.

*Avicennia marina* is one of mangrove plants and widely distribute in southeastern coast in China and its fruits are often picked and eaten by local residents [7]. Water extract of the fruits has traditionally been used for the treatments of colds, larynx and dysentery [8]. And the fruits have the effects of releasing inflammatory and dieresis as a vegetable directly [9]. Thus the fruits of *A. marina* reasonably meet the interests of modern people’s healthy food habits. Continuation of our study on searching for more bioactive molecules from the plant had led to the isolation of one new triterpenoid saponin (**1**) and 29 known compounds from the fruits of *A. marina*. Pharmacological experiments showed that the new triterpenoid saponin had antitumor activity and most of the known compounds had potential antioxidant activity.

## Results and Discussion

### Structures Identification of Isolated Compounds

With various chromatographic methods, 30 compounds were isolated from the EtOAc fraction and *n*-BuOH fraction of the fruits of *A. marina* ethanol extract. With the basis spectroscopic data and comparison with literature data, these compounds were identified as ilekudinoside B (**2**) [10], ilekudinoside C (**3**) [11], caffeic acid (**4**) [12], ferulic acid (**5**) [13], 4-hydroxycinnamic acid (**6**) [14], *p*-hydroxybenzoic acid (**7**) [15], 4-ethylcatechol (**8**) [16], 3,4,5-trimethoxybenzoic acid (**9**) [17], avicennone E (**10**) [18], avicennone D (**11**) [18], glechomol C (**12**) [19], *trans*-1,3-bis(3′,4′-dihy-droxyphenyl)-1-butene (**13**) [20], (*E*)-3-(3′-hydroxybut-1-enyl)-2,4,4-trimethylcyclohexa-2,5-dienone (**14**) [21], 3-(3′-oxobutyl)-2,4,4-trimethylcyclohexa-2,5-dienone (**15**) [22], 3-(3′-hydroxybutyl)-2,4,4-trimethylcyclohexa-2,5-dienone (**16**) [22], acteoside (**17**) [23], calceolarioside A (**18**) [24], campneoside I (**19**) [25], [*β*-D-glucopyranose] [3-*O*-(6-deoxy-*α*-l-mannopyranosyl)] [4-(2*E*)-3-(3,4-dihydrox-yphenyl)-2-propenoate] (**20**) [23], 6″-*O*-acetylacteoside(**21**) [26], crenatoside (**22**) [27], decaffeoylacteoside (**23**) [26], flavoyadorinin B (**24**) [28], marinoid D(**25**) [29], 10-*O*-(*trans*-feruloyl) geniposidic acid (**26**) [30], chlorogenic acid (**27**) [31], neochlorogenic acid(**28**) [31], cleroindicin E (**29**) [32], quinic acid ethyl ester (**30**) [33], and a new compound, 6′-*O*-(*n*-butanol) ilekudinoside B ester (**1**).

**Fig. 1:**
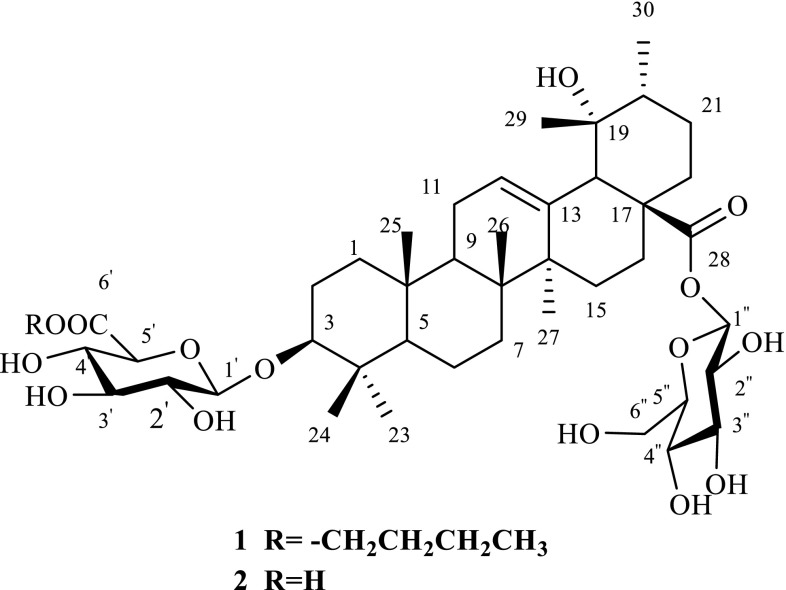
Structures of **1** and **2**

**Fig. 2:**
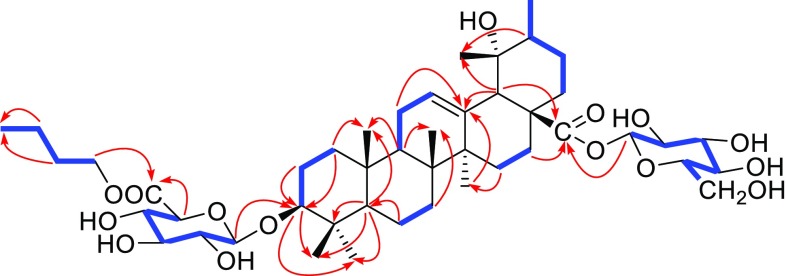
Key ^1^H-^1^H COSY (blue line) and HMBC (red arrow) correlations of compound **1**

**Fig. 3:**
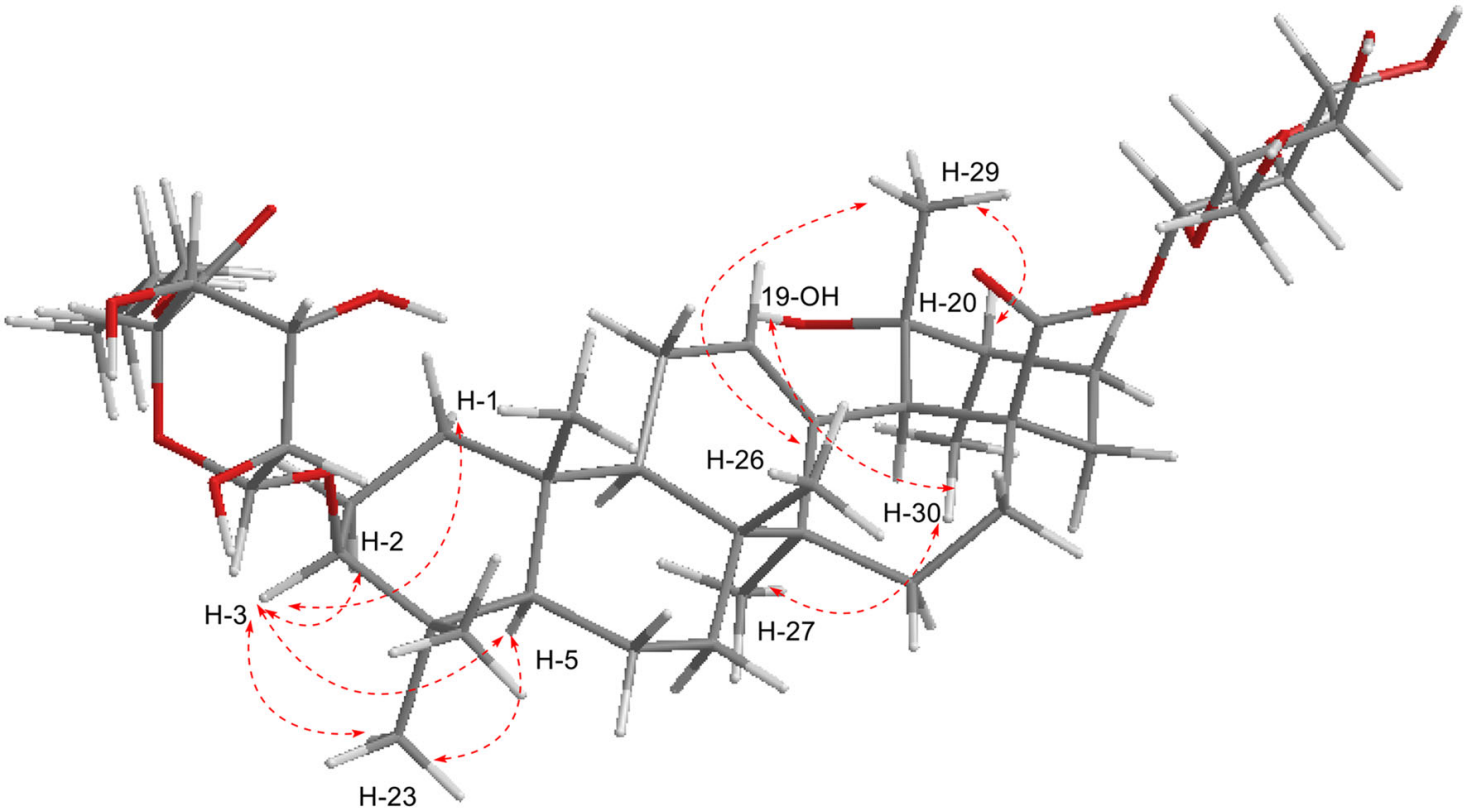
Key ROESY (red arrow) correlations of compound **1**

Compound **1** was obtained as white amorphous powder. Its molecular formula was determined to be C_46_H_74_O_15_ on the basis of its HRESIMS (m/z 889.4921 [M + Na]^+^, calcd C_46_H_74_O_15_Na, 889.4920), suggesting 10 degrees of unsaturation. The IR absorption bands at 3430 and 1735 cm^−1^ implied the presence of the hydroxyls and carboxyl groups, respectively. The ^1^H NMR spectrum of **1** exhibited signals for seven methyl groups at *δ*_H_ 1.27 (s), 1.07 (s), 0.97 (s), 0.85 (s), 0.75 (s), 0.66 (s) and 0.85 (d, *J *= 6.0 Hz) and an olefinic proton at *δ*_H_ 5.16 (s), which was in conformity with the appearance of seven methyls (*δ*_C_ 27.6, 16.3, 15.2, 16.4, 23.8, 26.4 and 16.3) and one olefinic signal (*δ*_C_ 127.1) in its ^13^C NMR data (Table 1). In addition, the presence of two anomeric carbon at *δ*_C_ 105.1 and 94.1, as well as other oxygenated carbon signals from *δ*_C_ 60.7 to 77.6 and a carboxyl at *δ*_C_ 169.1 in the ^13^C NMR spectrum, indicated that there existed sugar moiety. These data, in association with the literature investigation, suggested that compound **1** might have a structure related to that of ilekudinoside B (Fig. 1), except for the appearance of three methylenes (*δ*_C_ 64.1/*δ*_H_ 4.09, *δ*_C_ 30.1/*δ*_H_ 1.55, *δ*_C_ 18.5/*δ*_H_ 1.33) and one methyl (*δ*_C_ 13.6/*δ*_H_ 0.87), which were identified to *n*-butanol, and supported by the ^1^H–^1^H COSY correlations of 4.09/1.55/1.33/0.87 (Fig. 2) [34]. Comparison of the ^13^C NMR data of **1** with those of ilekudinoside B revealing the upfield shift (− 3.1 ppm) for C-6′, assumed that the additional *n*-butanol was attached to C-6′. The assumption was further supported by the HMBC correlation from H-1′″ (*δ*_H_ 4.09) to C-6′ (*δ*_C_ 169.1) (Fig. 2). The sugar analysis by GC after acid hydrolysis afforded d-glucose and d-glucuronic acid as component monosaccharides. And the coupling constants of the anomeric protons [*δ*_H_ 4.29 (d, *J* = 7.8 Hz, H-1′), 5.15 (d, *J* = 7.8 Hz, H-1″)] suggested *β*-pyranosyl configuration for both d-glucose and d-glucuronic acid moieties [35]. Moreover, in the ROESY spectrum, the ROESY correlation of *δ*_H_ 3.04 (H-3) with 0.72 (H-5) (Fig. 3) suggested that the configuration of H-3 was the *β* orientation, due to *α* oriented H-5 from the biosynthesis of ursane-type triterpene [36]. Meantime the ROESY correlation of *δ*_H_ 3.83 (19-OH) with 0.85 (30-H) suggested the configuration of 19-OH was *α* orientation (Fig. 3). Therefore, **1** was elucidated as 6′-*O*-(*n*-butanol) ilekudinoside B ester.

**Table 1:** ^1^H (500 MHz) and ^13^C NMR (125 MHz) data of **1** and ilekudinoside B in DMSO-*d*_6_

No.	**1**		Ilekudinoside B		No	**1**		Ilekudinoside B	
	*δ*_H_ (*J*, Hz)	*δ*_C_	*δ*_H_ (*J*, Hz)	*δ*_C_		*δ*_H_ (*J*, Hz)	*δ*_C_	*δ*_H_ (*J*,Hz)	*δ*_C_
1	0.87, m	38.3	0.87, m	38.3	1′	4.29 (d, 7.8)	105.6	4.15 (d, 7.7)	105.4
	1.49		1.47						
2	1.15	25.8	1.15	25.8	2′	3.00	73.6	3.01	74.1
	1.66, m		1.65, m						
3	3.04 (d, 4.2)	88.3	3.03 (d, 4.4)	88.0	3′	3.17	76.0	3.07	76.8
4		38.7		38.8	4′	3.31	71.4	3.25	72.3
5	0.72, m	55.0	0.70, m	55.1	5′	3.68 (d, 9.7)	75.3	3.59 (d, 11.5)	76.7
6	1.27, m	17.9	1.23, m	17.9	6′		169.1		172.2
	1.43, m		1.43, m						
7	1.22	32.6	1.15	32.6	1′′	5.15 (d, 7.8)	94.1	5.16 (d, 8.0)	94.1
	1.41, m		1.42, m						
8	1.55, m	39.5		39.5	2′′	3.08	72.3	3.09	73.9
9	1.55, m	46.7	1.54, m	46.7	3′′	3.12	77.6	3.29	79.2
10		36.2		36.2	4′′	3.11	69.5	3.24	69.5
11	1.84, m	23.2	1.90	23.2	5′′	3.17	76.7	3.30	77.6
12	5.16	127.1	5.17	127.1	6′′	3.43, 3.59	60.7	3.43, 3.59	60.6
13		138.2		138.2	1′″	4.09, m	64.1		
14		41.1		41.1	2′″	1.55, m	30.1		
15	0.84, m; 1.74	28.1	0.70, m; 1.76	28.1	3′″	1.33, m	18.5		
16	1.49, 2.51	25.1	1.51, 2.94	25.1	4′″	0.87, m	13.6		
17		47.8		47.4					
18	2.35, s	53.2	2.36, s	53.2					
19	3.83, s	71.7	3.83, s	71.7					
20	1.25, m	41.3	1.23, m	41.3					
21	1.14,	25.7	1.15	25.5					
	1.63		1.65						
22	1.46, m	36.7	1.46, m	36.7					
	1.61		1.64						
23	0.97, s	27.6	0.97, s	27.7					
24	0.75, s	16.3	0.76, s	16.4					
25	0.85, s	15.2	0.87, s	15.2					
26	0.66, s	16.4	0.67, s	16.5					
27	1.27, s	23.8	1.27, s	23.8					
28		175.6		175.6					
29	1.07, s	26.4	1.09, s	26.5					
30	0.85 (d, 6.0)	16.3	0.84 (d, 6.6)	16.3

### Anticancer Activity

**Fig. 4:**
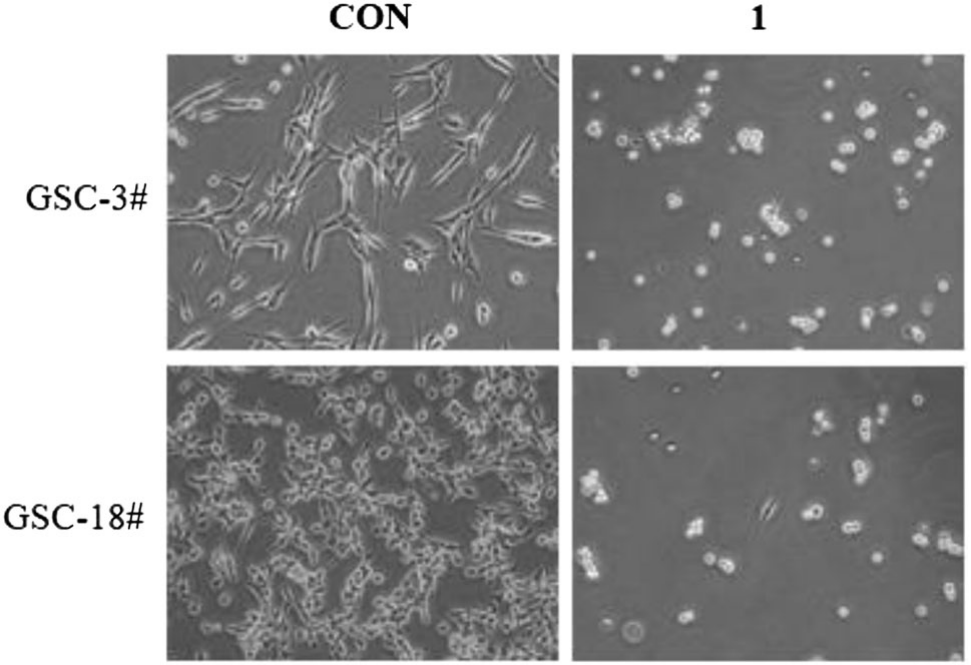
Compound **1** against human glioma stem cells by phenotypic screening at 20 μg/mL

**Fig. 5:**
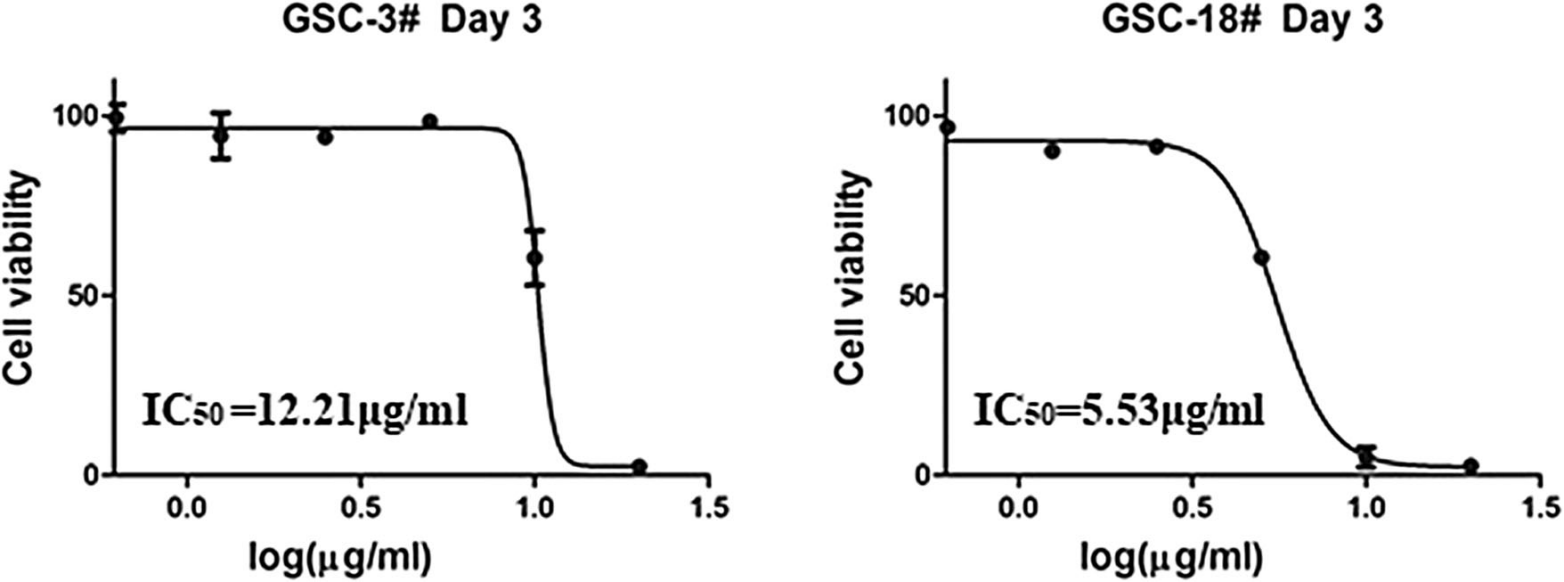
The IC_50_ value for compound **1** against human glioma stem cell lines

All compounds were evaluated for their bioactivities against two human glioma stem cell lines (GSC-3# and GSC-18#) by the cell viability assay and phenotypic screening. The results showed that compound **1** exhibited the moderate cytotoxicity against GSC-3# and GSC-18# at the concentration of 20 μg/mL (Fig. 4), and the IC_50_ values were 12.21 and 5.53 μg/mL, respectively (Fig. 5).

### Antioxidant Activity

DPPH is a widely-used stable free radical to evaluate antioxidant activity of bioactive. In this present assay, the free radical scavenging activities of the polyphenols and phenylethanoid glycosides were evaluated and compounds **4, 5, 8, 18–23, 27, 28** showed potent scavenging activities with IC_50_ values from 0.36 to 13.07 μg/mL. Especially compounds **4, 8, 18,** and **21** exhibited well scavenging capacities than vitamin C in the experiment (Table 2).

**Table 2:** DPPH scavenging activity and yield of antioxidant constituents of the fruit of *A. marina*

Compound	V_c_	4	5	8	18	19	20	21	22	23	27	28
IC_50_ (μg/mL)	1.95	0.36	2.99	1.15	4.07	8.84	12.54	4.34	8.44	13.07	8.95	5.78
Yield(mg/kg)		2.06	0.35	0.24	1.09	2.32	1.83	11.30	4.43	1.05	2.52	2.54

## Experimental Section

### General Experimental Procedures

1D and 2D NMR spectra were recorded on Bruker DRX-500 spectrometers using TMS as an internal standard. Chemical shifts (*δ*) were expressed in ppm with reference to the solvent signals. Optical rotations were measured on a JASCO P-1020 polarimeter. IR spectra were determined on a Bruker FT-IR Tensor-27 infrared spectrophotometer with KBr disks. UV spectra were detected on a SHMADZU UV-2401PC spectrometer. MS and HRMS analysis were carried out on Waters Xevo TQS and Waters AutoSpec Premier P776 mass spectrometers, respectively. Semipreparative HPLC was performed on a Waters 600 with a COSMOSIL C18 (10 × 250 mm) column. Silica gel (100–200 and 200–300 mesh, Qingdao Marine Chemical Co., Ltd., People’s Republic of China), and MCI gel (75–150 μm, Mitsubishi Chemical Corporation, Tokyo, Japan) were used for column chromatography. Fractions were monitored by thin-layer chromatography (TLC) (GF254, Qingdao Marine Chemical Co., Ltd.), and spots were visualized by 10% sulfuric acid ethanol solution.

### Plant Materials

The fresh fruits of *A. marina* were collected in Beibu Gulf of Guangxi province, China, and identified by Dr. Ya-Ping Liu, Kunming Institute of Botany, CAS. A voucher specimen (No. 20170402) has been deposited in the Kunming Institute of Botany, Chinese Academy of Sciences, Kunming, China.

### Extraction and Isolation

The fresh fruits of *A. marina* (14.2 kg) were extracted with 95% EtOH (40 L) three times under ambient temperature. After removing the solvent in vacuum, the residue (300 g) was suspended in H_2_O and extracted successively with petroleum ether (PE), ethyl acetate (EtOAc) and *n*-butanol (*n*-BuOH) respectively. After evaporation of the solvent in reduced pressure, the PE fraction (12 g), EtOAc fraction (18 g) and *n*-BuOH fraction (89 g) were obtained. The EtOAc fraction were separated by C18 silica gel column eluting with CH_3_OH/H_2_O (from 40:60 to 100:0, v/v) to yield four fractions (Fr-1 to Fr-4). Fr-4 was purified by C18 silica gel column eluting with CH_3_OH/H_2_O (10:90, v/v) to afford compound **7** (4 mg) and the residue, then the latter was further purified over HPLC using the mobile phase of 30% CH_3_CN/H_2_O and compounds **10** (2.0 mg) and **11** (2.4 mg) were obtained. Fr-3 was separated by silica gel column eluting with CHCl_3_/CH_3_OH (8:1, v/v) to provide compounds **4** (10 mg) and **5** (12 mg) and fractions Fr-3-1, Fr-3-2 and Fr-3-3. Fraction Fr-3-1 then was separated by silica gel column eluting with PE/Acetone (3:1, v/v) to afford compound **9** (60 mg). Fraction Fr-3-2 were further purified by HPLC using the mobile phase of 33% CH_3_CN/H_2_O to give compounds **8** (18 mg) and **6** (16.9 mg). Compounds **14** (12 mg) and **16** (3.8 mg) from fraction Fr-3-3 by silica gel column and eluted by PE/acetone (5:1, v/v). Fr-2 was divided into two fractions (Fr-2-1 and Fr-2-2) by Sephadex LH-20 column, eluting with 100% CH_3_OH. And then Fr-2-1 afforded compound **15** (2.8 mg) by silica gel column eluting with PE/acetone (2:1, v/v). Fr-2-2 was purified by silica gel column eluting with CH_3_Cl/acetone (2:1, v/v) to afford compound **12** (10 mg). Compound **13** (2.5 mg) were obtained from Fr-1 by HPLC eluting with 20% CH_3_CN/H_2_O.

The *n*-BuOH fraction was separated by C18 silica gel column which eluted by CH_3_OH/H_2_O (from 10:90 to 100:0, v/v) to yield eight fractions (Fr-I to Fr-VIII). Fr-VIII was divided into three subfractions (Fr-VIII-1 to Fr-VIII-3) by silica gel column gradually eluting with CH_3_Cl/CH_3_OH (10:1, 5:1, 0:1, v/v). And then fraction Fr-VIII-3 was separated by Sephadex LH-20 CC eluting with methanol to afford compound **1** (290 mg) and the residue. Compound **3** (24.3 mg) was obtained by silica gel column eluting with CH_3_Cl/CH_3_OH (5:1, v/v) from the residue. Compound **2** (3.8 g) were purified from Fr-VIII-2 by silica gel column eluting with CH_3_Cl/CH_3_OH (4:1, v/v). Fr-VIII-1 was purified by silica gel column eluting with CH_3_Cl/CH_3_OH (3:1, v/v) to afford **18** (15.5 mg). Compounds **19** (33.0 mg) and **20** (161 mg) were obtained by silica gel column eluting with CH_3_Cl/CH_3_OH (5:1, v/v) and then HPLC with 40% CH_3_CN/H_2_O as the mobile phase from Fr-VII. Fr-VI was divided into subfractions Fr-VI-1 and Fr-VI-2. And then compounds **21** (24.2 mg) and **23** (26 mg) were obtained from Fr-VI-1 by HPLC eluting with 45% CH_3_CN/H_2_O, and Fr-VI-2 eluting with 45% CH_3_CN/H_2_O to afford compounds **22** (63 mg) and **24** (2.2 mg). Fr-V was separated by silica gel column eluting with CH_3_Cl/CH_3_OH (3:1, v/v) and then purified by HPLC eluting with 33% CH_3_CN/H_2_O to afford compounds **25** (20 mg) and **26** (7.2 mg). Fr-IV was purified by silica gel column using the mobile phase of CH_3_Cl/CH_3_OH (3:1, v/v) to afford compound **17** (162 mg). Fr-III was further divided into two subfractions (Fr-III-1 and Fr-III-2). Compounds **27** (15 mg) and **28** (35.9 mg) were obtained from Fr-III-1 by HPLC eluting with 23% CH_3_CN/H_2_O. Fr-III-2 was purified by silica gel column eluting with CH_3_Cl/CH_3_OH (8:1, v/v) to afford compounds **29** (28.4 mg) and **30** (27.7 mg).

*6′*-*n*-*butanol, ilekudinoside B ester* (**1**) White amorphous powder; $[\alpha ]_{\text{D}}^{20}$ − 20.1 (*c* 1.30, MeOH)]; IR (KBr) *ν*_max_ 3430, 2932, 1735, 1070 cm^−1^; UV (MeOH) *λ*_max_ (log *ε*) 206 (4.5) nm; HRESIMS m/z 889.4921 ([M + Na]^+^) (calcd for C_46_H_74_O_15_Na, 889.4920); ^1^H and ^13^C NMR spectroscopic data, see Table 1.

### Acid Hydrolysis of Compound **1**

As previously described [37] with some minor modifications, compound **1** (10 mg) was hydrolyzed with 2 M HCl/dioxane (1:1, 4 mL) under reflux for 8 h. After partitioned with CHCl_3_ (2 mL × 3), the aqueous layer was neutralized with 2 M NaOH and then dried to give a monosaccharide mixture. A solution of the sugar mixture in pyridine (2 mL) was added to l-cysteine methyl ester hydrochloride (about 1.5 mg) and kept at 60 °C for 1 h. Then trimethylsilylimidazole (1.5 mL) was added to the reaction mixture. After kept at 60 °C for 30 min, the reaction mixture was immediately subjected to GC analysis, run on a Shimadzu GC-14C gas chromatography equipped with an H_2_ flame ionization detector. The column was a 30 m × 0.32 mm i.d. 30QC2/AC-5 quartz capillary column with the following conditions: column temperature: 180–280 °C; programmed increase: 3 °C/min; carrier gas: N_2_ (1.0 mL/min); injector and detector temperature: 250 °C; injection volume: 4 μL; and split ratio: 1/50. By comparing with the retention time of the authentic sugars in the form of derivatives under the same condition, the sugar moieties of compound **1** was determined to be d-glucose (*t*_R_: 22.895 min), D-glucuronic acid (*t*_R_: 23.958 min) by compare with standard d-glucose (*t*_R_: 22.806 min) and D-glucuronic acid (*t*_R_: 23.907 min).

### Anticancer Activity

GSC-3# and GSC-18# were human glioma stem cell lines that were established by Kunming institute of zoology from two human glioblastoma multiform samples. The glioma stem cell was cultured in serum-free medium DMEM F12 supplied with 1xB27 and 50 ng/mL EGF, BFGF and 1% penicillin/streptomycin. The glioma stem cells were seeded in the laminin pre-coating dishes and cultured in 37 °C, 5% CO_2_ incubator. Cell viability assay was performed by the MTS method as previously described. GSCs were digested and counted, seeded in laminin pre-coating 96-well-plate with 20000 cells/well. The compounds were added with a serial gradient concentration (20, 10, 5, 2.5, 1.25, 0.625 μg/mL) and cultured in cell incubator for 72 h. MTS reagent was diluted 1:5 with fresh medium and mixed well. The old medium was removed and subsequently the fresh medium was added with 100 μL/well. The cells were incubated for 1.5 h. Absorbance was measured by Hybrid Reader (BioTek synergy H1) at 490 nm. The cell viability was evaluated by percentage compared with DMSO control group. The half-maximal inhibitory concentration (IC_50_) was measured and calculated by Graph Pad Prism 5 software.

### DPPH Free Radical Scavenging Activity

The antioxidant activity of compounds was evaluated using the DPPH free radical scavenging assay. The procedure used is an adaptation of those previously [38]. Samples were dissolved to various concentrations (25, 15, 5, 2, 0.5 μg/mL) with methanol. In this assay, reaction mixtures containing a methanolic solution of 0.1 mmol/L DPPH (160 μL) and serial dilutions of test samples (40 μL) were placed in a 96-well-plate. MeOH was used as a negative control and vitamin C was used as a positive control. The reactive mixtures were incubated at 37 °C for 30 min in darkness. The absorbance of the mixtures at 517 nm was measured by microplate reader. The scavenging activity (%) was determined by the following equation:

$${\text{DPPH scavenging activity }}\left( \% \right)\, = \,\left( { 1- {\text{A}}_{\text{x}} /{\text{A}}_{\text{o}} } \right)\, \times \, 100\% .$$

Percent inhibition was plotted against concentration, and the equation for the line was used to obtain the IC_50_ value.

## Electronic supplementary material

Below is the link to the electronic supplementary material.

1D and 2D NMR spectra, HRESIMS, IR and UV spectra and Optical rotation of compound **1** are available as Supplementary Information. Supplementary material 1 (DOCX 978 kb)
