# Supplementary material for: Antitumor Triterpenoid Saponin from the Fruits of Avicennia marina
Source: Nat Prod Bioprospect. 2018 May 25;8(5):347–53. doi: 10.1007/s13659-018-0167-9 (PMC6109444; doi:10.1007/s13659-018-0167-9)
Supplement: Supplementary file 1 — 1D and 2D NMR spectra, HRESIMS, IR and UV spectra and Optical rotation of compound 1 are available as Supplementary Information. Supplementary material 1 (DOCX 978 kb) [file 13659_2018_167_MOESM1_ESM.docx]

## Supplementary Data

## Antitumor Triterpenoid Saponin from the Fruits of *Avicennia marina*

Xiong-Wu Yang^1,3^, Zhi Dai ^2,3^, Bei Wang^1,3^, Ya-Ping Liu^1,4^, Xu-Dong Zhao^2,*^, Xiao-Dong Luo^1,4,^*

1 *State Key Laboratory of Phytochemistry and Plant Resources in West China, Kunming Institute of Botany, Chinese Academy of Sciences, Kunming 650201, People’s Republic of China*  2 *Key Laboratory of Animal Models and Human Disease Mechanisms, Kunming Institute of Zoology, Chinese Academy of Sciences, Kunming 650223, People’s Republic of China*  3 *University of Chinese Academy of Sciences, Beijing 100049, People’s Republic of China* 4 *Yunnan Key Laboratory of Natural Medicinal Chemistry, Kunming, 650201, P. R. China*

* Corresponding authors:

*E-mail address*: [xdluo@mail.kib.ac.cn](mailto:xdluo@mail.kib.ac.cn) (X. D. Luo); [zhaoxudong@mail.kiz.ac.cn](mailto:zhaoxudong@mail.kiz.ac.cn) (X. D. Zhao).

**Table of Contents**

| No. | Contents |
| --- | --- |
| 1 | **Fig. S1** ^1^H NMR spectrum of compound **1** in DMSO-*d*_6_ |
| 2 | **Fig. S2** ^13^C NMR spectrum of compound **1** in DMSO-*d*_6_ |
| 3 | **Fig. S3** COSY spectrum of compound **1** in DMSO-*d*_6_ |
| 4 | **Fig. S4** HSQC spectrum of compound **1** in DMSO-*d*_6_ |
| 5 | **Fig. S5** HMBC spectrum of compound **1** in DMSO-*d*_6_ |
| 6 | **Fig. S6** ROESY spectrum of compound **1** in DMSO-*d*_6_ |
| 7 | **Fig. S7** HRESIMS spectrums of compound **1** in DMSO-*d*_6_ |
| 8 | **Fig. S8** UV spectrum of compound **1** in Methanol |
| 9 | **Fig. S9** IR spectrum of compound **1** |
| 10 | **Fig. S10** Optical rotation of compound **1** |
| 11 | **Fig. S11** The GC spectra of standard D-glucose |
| 12 | **Fig. S12** The GC spectra of standard D-glucuronic acid |
| 13 | **Fig. S12** The GC spectra of the sugar moieties of compound 1 |


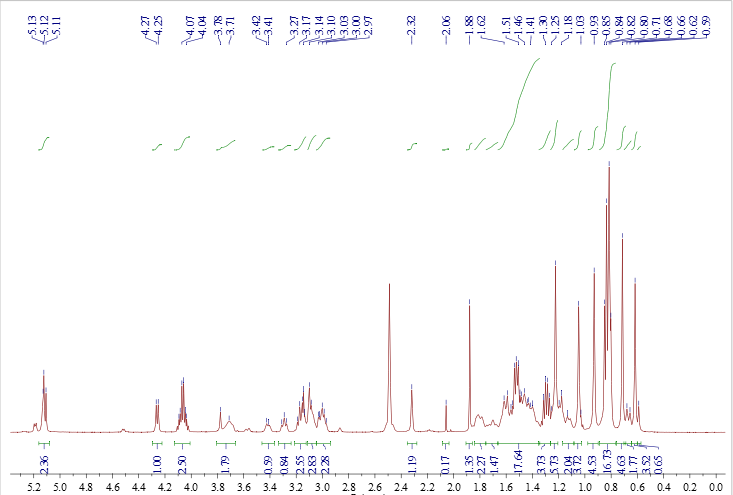


**Fig. S1** 1H NMR spectrum of compound **1** in DMSO-*d*_6_


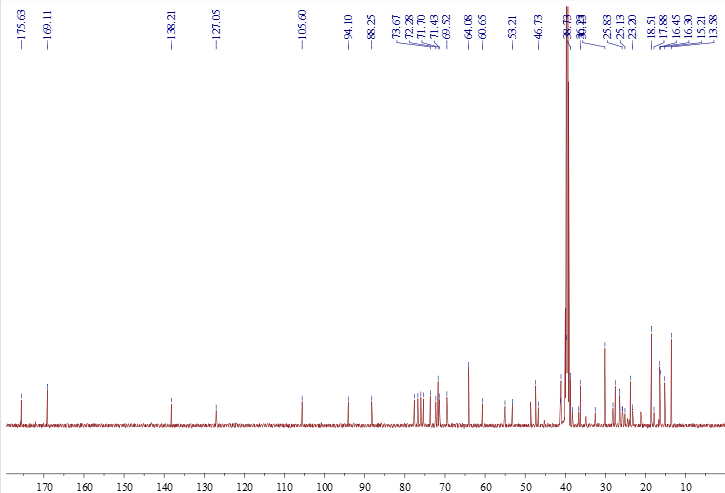


**Fig. S2** ^13^C NMR spectrum of compound **1** in DMSO-*d*_6_


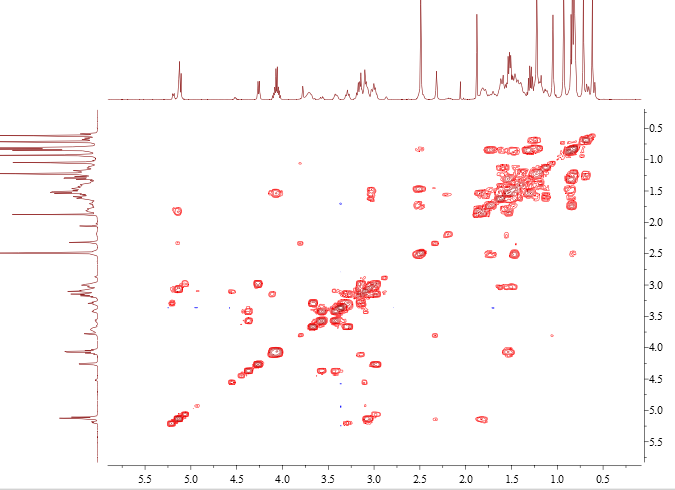


**Fig. S3** COSY spectrum of compound **1** in DMSO-*d*_6_


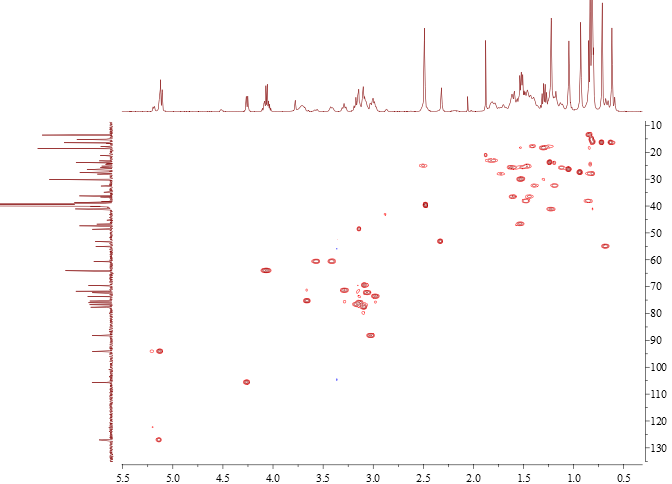


**Fig. S4** HSQC spectrum of compound **1** in DMSO-*d*_6_


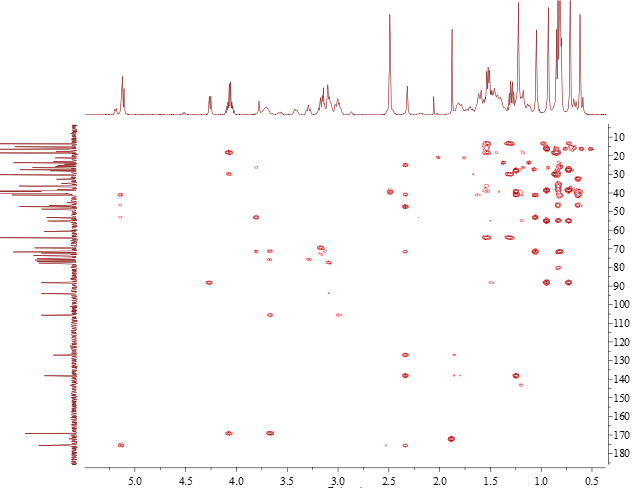


**Fig. S5** HMBC spectrum of compound **1** in DMSO-*d*_6_


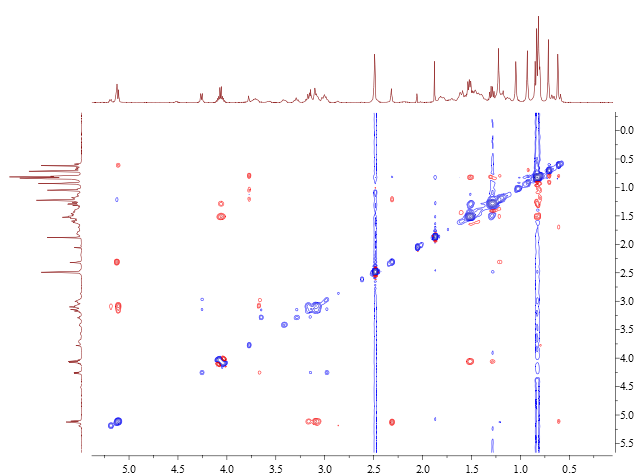


**Fig. S6** ROESY spectrum of compound **1** in DMSO-*d*_6_


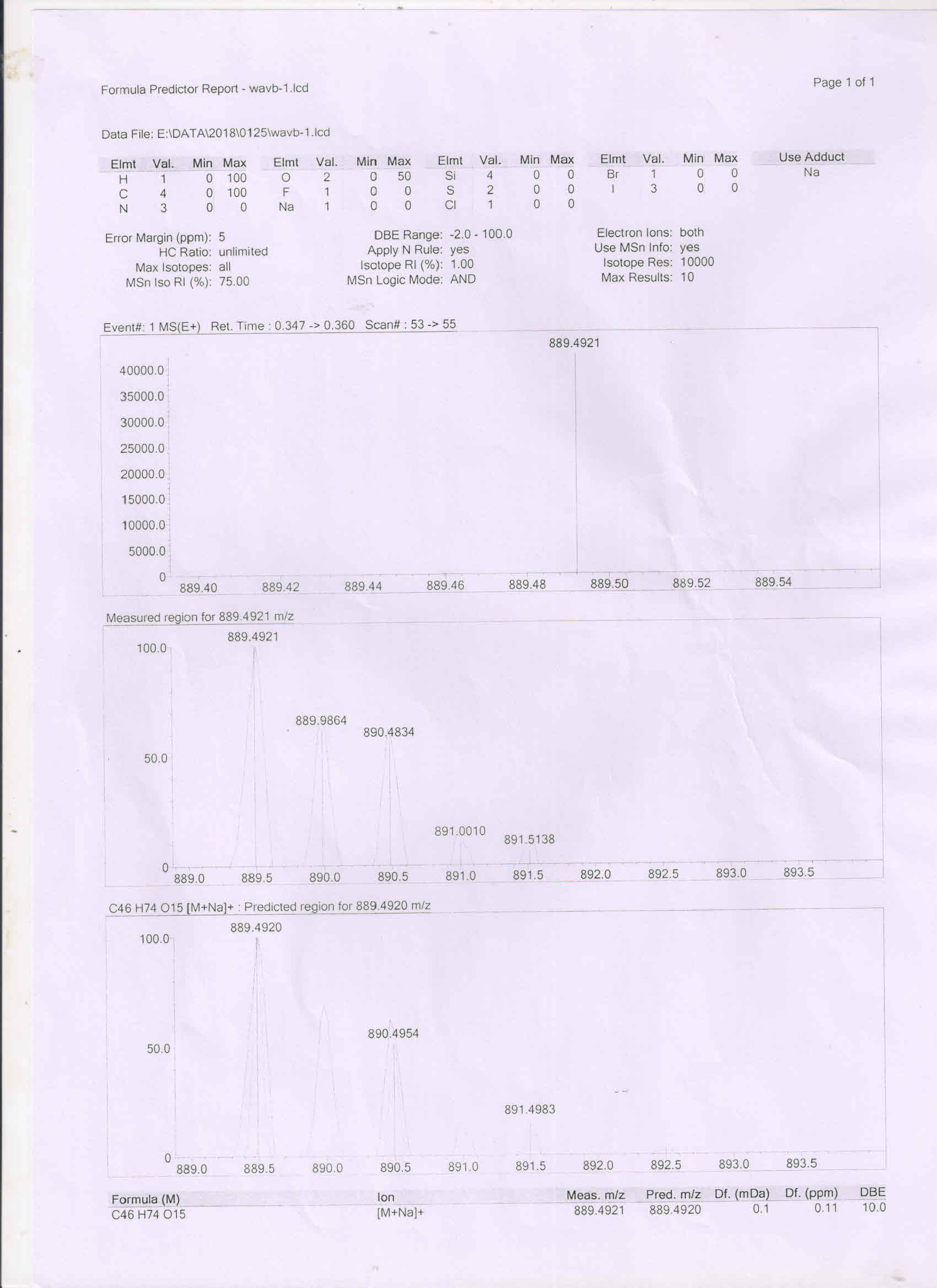


**Fig. S7** HRESIMS spectrums of compound **1** in DMSO-*d*_6_

**
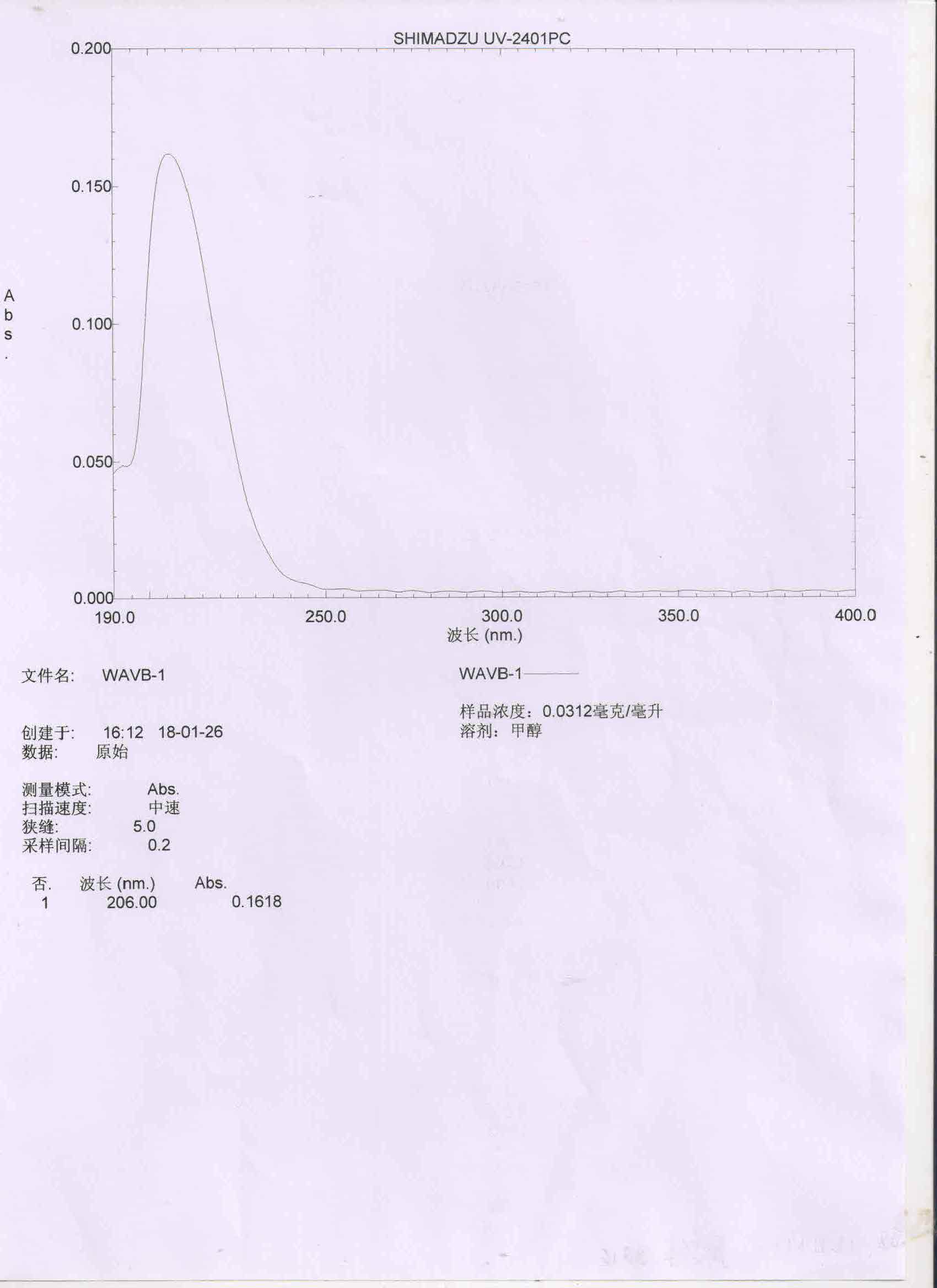
**

**Fig. S8** UV spectrum of compound **1** in Methanol


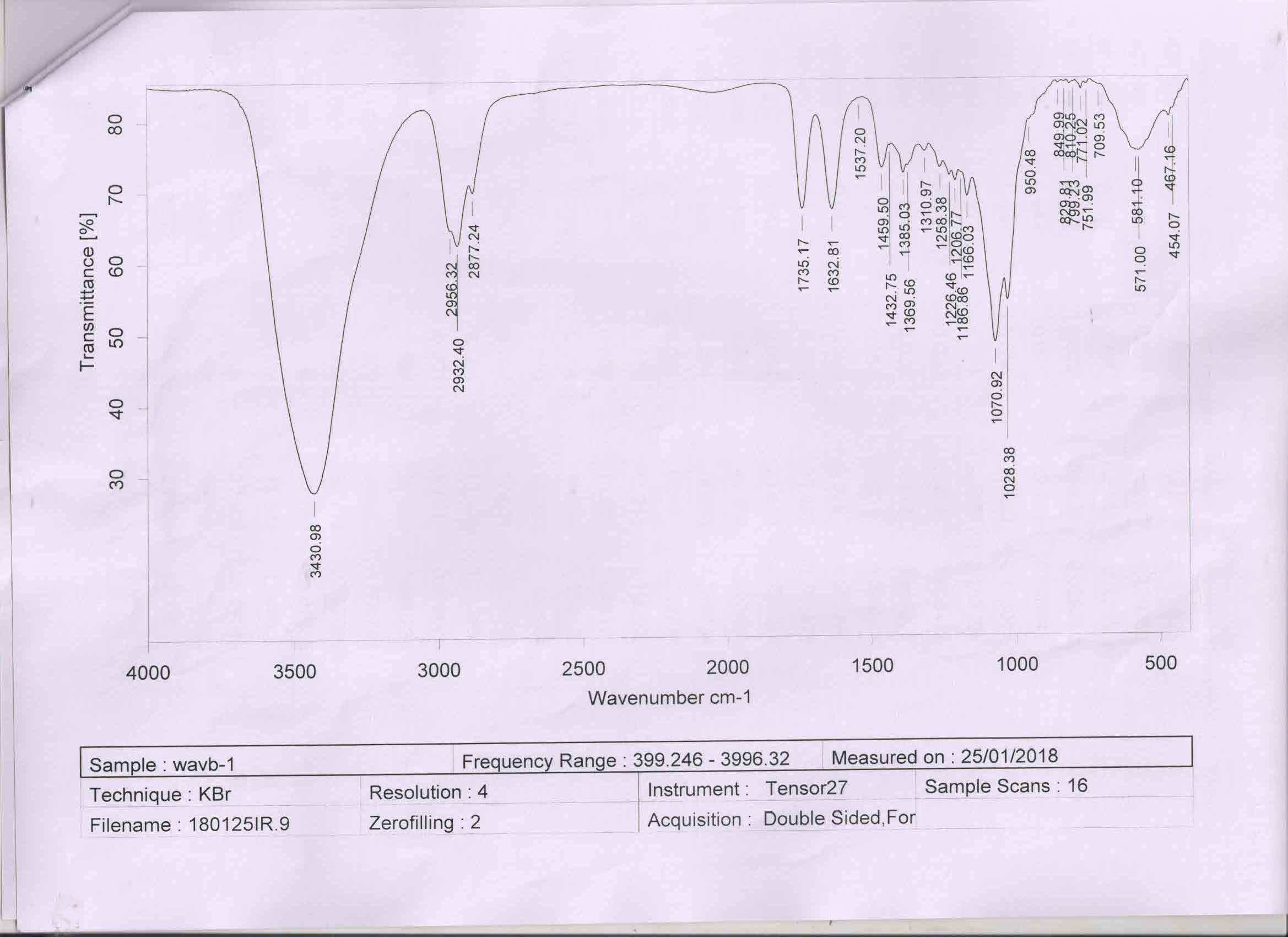


**Fig. S9** IR spectrum of compound **1**


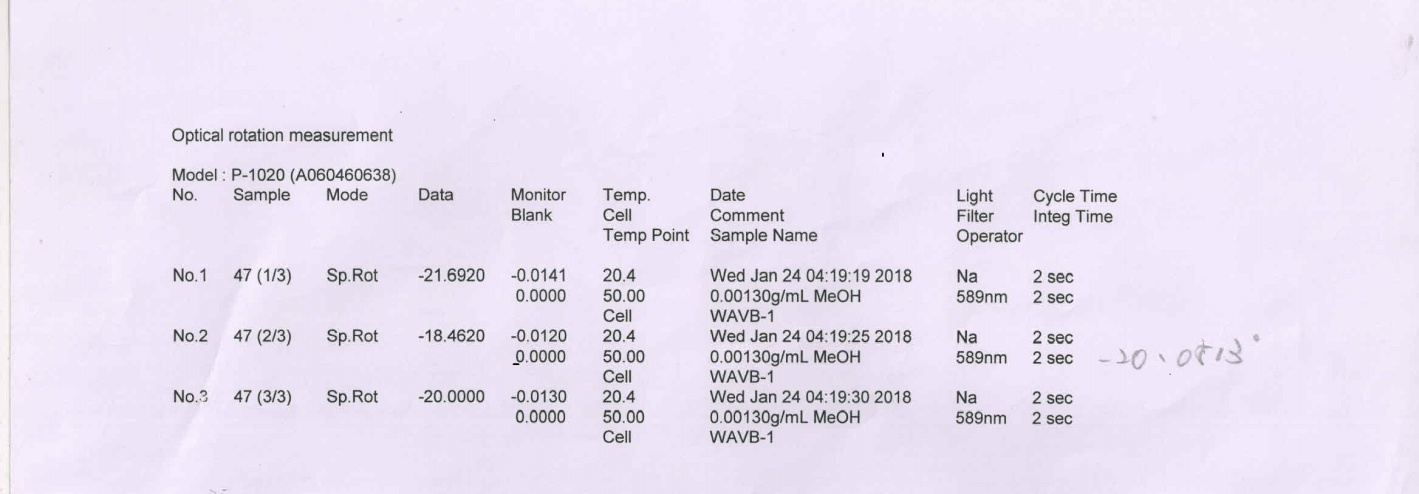


**Fig. S10** Optical rotation of compound **1**


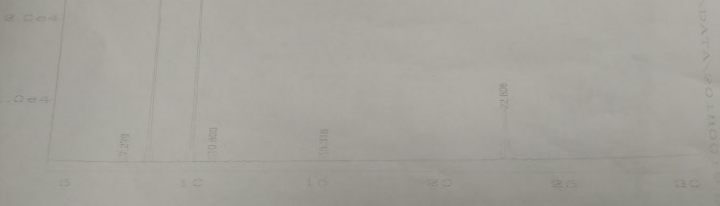


**Fig. S11** The GC spectra of standard D-glucose


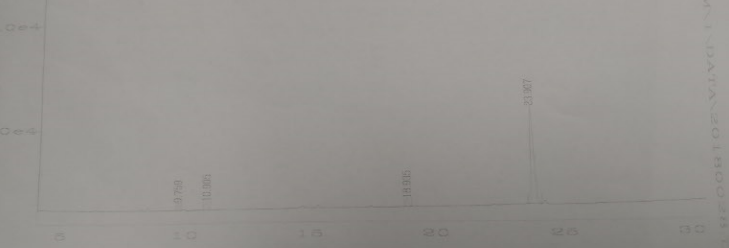


**Fig. S12** The GC spectra of standard D-glucuronic acid


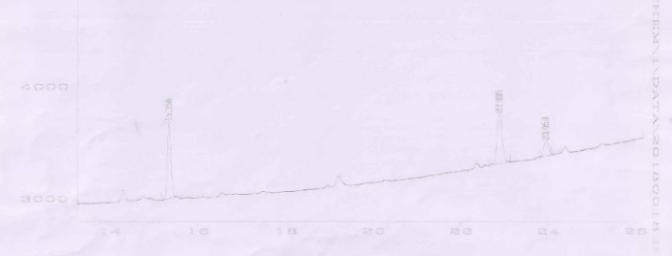


**Fig. S12** The GC spectra of the sugar moieties of compound 1
